# Beta‐amyloid is the most promising target for Alzheimer’s disease treatment

**DOI:** 10.1002/alz.084126

**Published:** 2025-01-03

**Authors:** Beka Solomon

**Affiliations:** ^1^ Tel Aviv university, Tel Aviv, Israel Israel

## Abstract

**Background:**

Amyloid filaments formation is a complex kinetic and thermodynamic process. The dependence of peptide polymerization on peptide–peptide interactions to form a *β*‐pleated sheet fibrils and the stimulatory influence of other proteins on the reaction suggest that amyloid formation may be subject to modulation

**Method:**

*In vitro* formation of β‐amyloid was induced by incubation of an aqueous solution of AβP (10 mg/ml) for 7 days at 37°C. The extent of β‐amyloid formation and disaggregation were monitored using a panel of well characterized mAbs raised against soluble AβP fragments. The aggregation of Aβ was measured by the ThT binding assay, in which the fluorescence intensity reflects the degree of β‐amyloid fibrillar aggregation.

**Result:**

The epitopes of the anti ‐aggregated antibodies were localized employing a library of filamentous phage displaying random combinatorial hexapeptides. Among 44 phages, the consensus sequence EFRH was carried by 40 clones. The EFRH (3‐6) of AβP located at the N‐terminal is exposed for antibody binding in both forms, either in solution or an aggregate, suggesting that he is involved in the aggregation process and acts as a regulatory site controlling both the prevention and the disaggregation process.

**Conclusion:**

Our pioneering data were recently confirmed by successful Aducanumab (3‐7) and Lecanemab (1‐16) antibodies that recognize the N terminus of Aβ and selectivity bind to aggregated Aβ species. Naturally occurring anti‐AβP antibodies have been found in human CSF and in the plasma of healthy individuals, from where Aducanumab was derived, but were significantly lower in **Alzheimer’s disease** patients.

